# Preparation of a liposomal delivery system and its *in vitro* release of rapamycin

**DOI:** 10.3892/etm.2015.2201

**Published:** 2015-01-22

**Authors:** ZHI-LIN MIAO, YING-JIE DENG, HONG-YANG DU, XU-BIN SUO, XIAO-YU WANG, XIAO WANG, LI WANG, LI-JIE CUI, NA DUAN

**Affiliations:** 1 Heart Center, The People’s Hospital of Liaoning Province, Shenyang, Liaoning 110016, P.R. China; 2 School of Pharmacy, Shenyang Pharmaceutical University, Shenyang, Liaoning 110016, P.R. China; 3 Dalian Medical University Graduate School, Dalian, Liaoning 116044, P.R. China

**Keywords:** atherosclerosis, rapamycin, liposomes, orthogonal design, *in vitro* release kinetics, reverse dialysis, targeted therapy

## Abstract

The aim of this study was to prepare a liposomal delivery system for rapamycin and study its *in vitro* release characteristics. The results may provide a foundation for the further development of a liposomal delivery system for rapamycin and the establishment of a new active treatment method targeted towards the cellular components of atherosclerotic plaques. The ethanol injection method was used to prepare rapamycin-containing liposomes. The formulation was optimized by orthogonal design, and the degree of rapamycin release by the liposomes was measured by the reverse dialysis method. Orthogonal testing showed that the optimum formulation had a phospholipid concentration of 4%, a phospholipid-cholesterol mass ratio of 8:1, a drug-lipid mass ratio of 1:20 and an aqueous phase pH of 7.4. Rapamycin-containing liposomes with an encapsulation efficiency of 82.11±2.13% were prepared, and the *in vitro* release of rapamycin from the liposomes complied with a first-order kinetic equation. In conclusion, the formulation was optimized, the prepared liposomes had a high rapamycin encapsulation rate and good reproducibility, and their *in vitro* release had a certain delayed-release effect.

## Introduction

Cardiovascular disease is a major disease that threatens human health, with the main pathological feature being atherosclerosis (AS). The identification of methods for suppressing intimal hyperplasia and delaying the progress of AS remains a worldwide challenge (1). There is currently no effective preventive measure for diseases with intimal plaque hyperplasia as the main pathological feature; in particular, there is no drug that directly targets the molecular components of blood-intimal atherosclerotic plaques.

Rapamycin and its derivatives can inhibit the proliferation of vascular smooth muscles (2). In certain research, rapamycin exhibited significant effects against restenosis occurring post-coronary intervention when used as a drug-coated stent (3). While a drug-coated stent is effective in the focal vessel, it is not able to act at non-stent covered sites or in other systemic diseases, such as AS, which cannot be treated with a stent (4). Moreover, rapamycin is a drug with poor solubility that is unstable in stomach acid, and has an oral bioavailability of only 14%. Therefore, it cannot play a systemic role through oral or intravenous administration methods (5).

In the present study, rapamycin was encapsulated into liposomes. In the future, these may be modified with an antibody or receptor, so that the liposomes are actively targeted via antigen-antibody or substrate-receptor combination towards the molecular components of atherosclerotic plaques, thus developing a receptor- or antibody-mediated active targeting administration system towards atherosclerotic plaques. Such a system could greatly increase the concentration of drug at the focal site, and provide a new prevention method with active targeting towards a variety of diseases involving AS.

In this study, the ethanol injection method was used to prepare a liposomal rapamycin-delivery system, and the formulation was optimized. Reverse dialysis was used to investigate the *in vitro* release characteristics of the rapamycin-containing liposomes, with the aim of exploring the mechanism, method and characteristics of *in vitro* release. The findings should lay a foundation for the further development of a liposomal delivery system for rapamycin, actively targeted towards the cellular components of atherosclerotic plaques.

## Materials and methods

### Preparation of rapamycin-containing liposomes

Various amounts of phospholipids (Shanghai Taiwei Pharmaceutical Co., Ltd., Shanghai, China), cholesterol (Tianjin Bodi Chemical Co., Ltd., Tianjin, China) and rapamycin (purity, 99.2%; Shanghai Qi Ao Chemical Science Co., Ltd., Shanghai, China) were dissolved in various amounts of absolute ethanol. Then, under stirring, the above mixture was slowly and uniformly injected into phosphate buffer (Tianjin Bodi Chemical Co., Ltd.), and the resulting mixture was stirred at 60°C. The ethanol was then removed by reduced-pressure evaporation, and the obtained crude liposome solution was then sequentially filtered through 0.8-, 0.45-, 0.22- and 0.1-μm membranes, five times each, for particle preparation. Finally, rapamycin-containing liposomes were obtained.

### Establishment of the method for the determination of rapamycin content

This was conducted using a Diamonsil C18 column (150×4.6 mm, 5 μm; Dikma Technologies Inc., Lake Forest, CA, USA) with Hitachi Pump L-2130 and UV Detector L-2400 (Hitachi, Tokyo, Japan). The mobile phase was methanol (chromatographic grade; Jiangsu Hanbang Technology Co., Ltd., Huai’an, China) and water (78:22), with a flow rate of 1.0 ml/min. The UV detection wavelength was 278 nm, the column temperature was 50°C and the injection volume was 20 μl

A 10.0 mg sample of rapamycin was precisely weighed into a 100-ml volumetric flask, dissolved in acetonitrile (chromatographic grade; Jiangsu Hanbang Technology Co., Ltd.) and diluted to the 100 ml mark, which gave a stock solution of rapamycin at the concentration of 100 mg/l. A precise amount of this stock solution was diluted with acetonitrile to form solutions with concentrations of 2.0, 5.0, 10.0, 20.0, 50.0 and 100.0 mg/l. The samples were analyzed according to the chromatographic conditions described above. Then, a linear regression plot was prepared of mass concentration (C) to peak area (A). The standard curve had the formula: A = 55307C − 9873.2 (r=1), indicating that the linear relationship of rapamycin was good in the range of 2 to 100 mg/l.

Into a 10-ml flask was added 0.5 ml blank liposome, followed by 0.5, 2.0 or 5.0 ml stock rapamycin solution. Methanol was used to break the liposomes and for dilution. The process was repeated three times, and sample solutions with concentrations of 5.0, 20.0 and 50.0 mg/l were obtained for injection and the calculation of recovery. The recovery rates were 99.73, 100.5 and 98.02%, respectively, with relative standard deviations of 0.24, 1.70 and 1.74 %, respectively.

### Determination of encapsulation efficiency (EE)

Sephadex G-50 microcolumn-centrifuging high-performance liquid chromatography (HPLC) was used to separate the liposomes and free rapamycin for the determination of EE (6,7). The specific steps were as follows: Sephadex G-50 (Bio-Rad, Hercules, CA, USA), which was fully swelled with distilled water, was placed into a 5-ml syringe, then centrifuged at 600 × g for 3 min, dehydrated and used to establish the Sephadex G-50 microcolumn. Following the addition of 0.5 ml rapamycin-containing liposomes to the top of the microcolumn, the column was centrifuged at 600 × g for 3 min and the separated liquid was collected. Then, 0.5 ml pH 7.4 buffer was continuously added and the column was centrifuged using the same method to elute the liposomes. The above process was repeated twice, and the eluent was collected. Methanol was used to break the liposomes and dilute to a volume of 10 ml prior to sample determination to calculate the concentration of rapamycin (C_1_) encapsulated inside the liposomes. Another 0.5 ml liposomes were directly diluted with methanol to the same extent, but were not subjected to microcolumn centrifugation, and sample determination was conducted to calculate the rapamycin concentration C_2_. EE was then calculated using the formula: EE (%) = C1/C2 × 100.

### Statistical methods

Experimental data were statistically processed using SPSS 12.0 software (SPSS, Inc., Chicago, IL, USA) and were expressed as mean ± standard deviation. The results of orthogonal experiments were analyzed by multivariate analysis of variance (MANOVA), while partial results were analyzed by ANOVA.

## Results

### Investigation of univariate factors of the rapamycin-containing liposome formulation

The phospholipid-cholesterol mass ratio, the drug-lipid ratio and the aqueous phase pH of the formulation were fixed; only the concentrations of phospholipid was changed to prepare liposomes with phospholipid concentrations of 1, 2, 3, 4 and 5%, with the aim of investigating the impact of phospholipid concentrations on the EE. The results demonstrated that in these experimental conditions, as the phospholipid concentration increased, the EE increased. However, excessively high phospholipid concentrations resulted in the aggregation of phospholipids, making it difficult for the rapamycin to be released.

The phospholipid concentration, drug-lipid ratio and the aqueous phase pH of the formulation were fixed; only the quantity of cholesterol was changed to prepare liposomes with phospholipid-cholesterol mass ratios of 15:1, 10:1, 8:1, 6:1, 4:1 and 3:1. The results indicated that the EE exhibited a trend of increasing initially and then decreasing as the amount of cholesterol incorporated into the liposomes increased. Cholesterol played the role of a regulating agent towards liposome membrane fluidity, and improved the drug EE and stability. However, excessive amounts of cholesterol competed for position in the phospholipid bilayer position with the liposoluble drug, namely rapamycin, resulting in decreased EE.

The phospholipid concentration, phospholipid-cholesterol mass ratio and the aqueous phase pH of the formulation were fixed, and the drug-lipid ratio was established at 1:10, 1:15, 1:20, 1:30 and 1:40 to prepare the liposomes. The results indicated that when the drug-lipid ratio was greater than 1:20, the EE was low.

The phospholipid concentration, phospholipid-cholesterol mass ratio and drug-lipid ratio of the formulation were fixed, and the ethanolic solution of the formulation was injected into phosphate buffer with a pH of 5.8, 6.5, 7.0, 7.4 or 8.0 to prepare liposomes. The results demonstrated that liposomes prepared at a pH of between 6.5 and 7.4 were stable and the EE was high, while flocculation or aggregation occurred under other pH conditions.

### Optimization of the rapamycin-containing liposome formulation by orthogonal design

Based on the investigation of single factors, the four factors, namely phospholipid concentration (factor A), phospholipid-cholesterol mass ratio (factor B), drug-lipid ratio (factor C) and aqueous phase pH (factor D) were selected, and three levels of each factor were designated for the orthogonal design, with the EE as the investigating indicator to screen the formulation. The orthogonal factors and levels are shown in Table I.

The L_9_ (3^4^) orthogonal table (8) was used and nine formulations were obtained, according to the above designs. Rapamycin-containing liposomes were prepared for EE determination. The orthogonal test results are shown in Table II, and the variance analysis is shown in Table III.

It can be determined by direct-viewing analysis of the extremum values (R) in Table II that the importance degrees of the factors were in the order: A>C>D>B, and the optimized formulation composition was A_3_B_2_C_3_D_1_, that is, the phospholipid concentration was 4%, the phospholipid-cholesterol ratio was 8:1, the drug-lipid ratio was 1:30 and the pH was 7.4.

The variance analysis indicates that factors A, C and D had significant impacts on the experimental results, and factor B was also significant. According to the F value, the impacts of various factors towards the test results were A>C>D>B, which is consistent with the results of direct-viewing analysis.

The optimized formulation had a drug-lipid ratio of 1:30; however, considering the amount of liposomal drug loading, a drug-lipid ratio of 1:20 was selected. As for factor C, the levels 2 and 3 were subjected to single-factor variance analysis, and the result was F<F_0.05_ (1,4)=7.71, indicating that the levels 2 and 3 of factor C were not statistically significant towards the experimental results. The final formulation was adjusted to A_3_B_2_C_2_D_1_, that is, the phospholipid concentration was 4%, the phospholipid-cholesterol ratio was 8:1, the drug-lipid ratio was 1:20 and the pH value was 7.4. The preparation and analysis of the above optimal formulation was repeated three times, and the EE of the rapamycin-containing liposomes was measured as 82.11±2.13%.

### Investigation of *in vitro* release

A previously described reverse dialysis method (9) was modified for the determination of the *in vitro* release of rapamycin from the liposomes.

**Figure 1:**
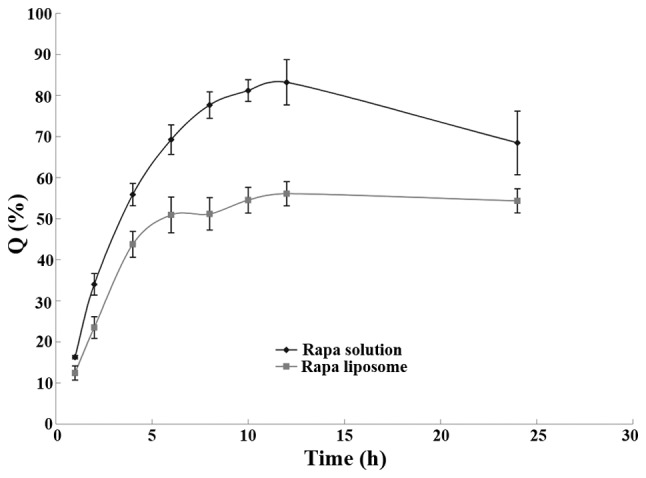
Release curves of rapamycin (rapa) solution and liposomes *in vitro*. Q, accumulative release rate.

In the reverse dialysis, 500 ml 20% ethanol was used as the release medium; 10 ml release medium was drawn and placed into a dialysis bag (diameter, 25 mm; trapping substances with a relative molecular mass of 12,000–14,000; Beijing Huamei Biotechnology Co., Ltd, Beijing, China). The dialysis bag was then clamped and attached on the paddle of a dissolution apparatus; 5 ml rapamycin-containing liposomes and 5 ml ethanol solution of rapamycin with the same drug content as the liposomes, were respectively dissolved in the dialysis vessel, with stirring at 37°C and 300 × g. Sampling of 100 μl liquid from the dialysis bag was conducted at 1, 2, 4, 6, 8, 10, 12 and 24 h for sample determination and calculation of the accumulative release rate. A release curve was drawn using time (t) as the abscissa, and the accumulative release rate (Q%) as the ordinate; the resulting curve is shown in Fig. 1.

It was found from the experiment that after 12 h, the amount of drug released gradually decreased with the extension of release time. Theoretically, the accumulative release of the drug should be maintained at a high level; however, at 37°C, rapamycin was unstable in aqueous solution and the drug content was reduced (10). Thus, it was necessary to consider the degradation of rapamycin in the release medium.

**Figure 2:**
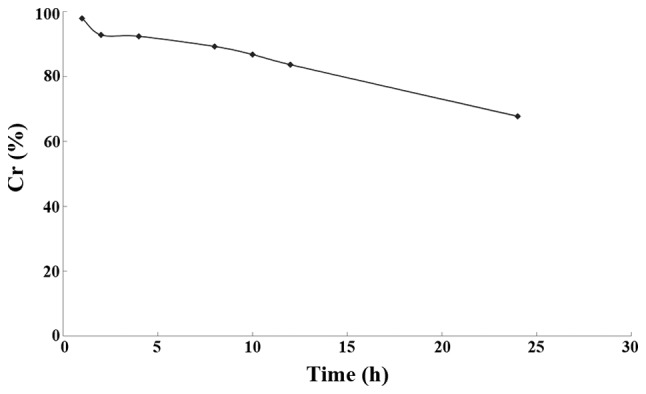
Degradation kinetic curve of rapamycin solution in release medium (37°C). Cr, residual percentage.

The 20% ethanolic solution of rapamycin was placed in a 37°C water bath, and sampled at 0, 1, 2, 4, 8, 10, 12 and 24 h, respectively, for the determination of rapamycin concentration (Ct) by HPLC at different time points. The ratio Ct/Co, where Co is the rapamycin concentration at 0 h, was used to calculate the residual percentage Cr, with the time t as the abscissa and Cr as the ordinate. The resulting curve is shown in Fig. 2.

The results demonstrated that rapamycin was unstable in the release medium; after 24 h, the drug content was 67.72% of the initial content. Linear fitting plots for zero-, first- and second-order models were drawn to study the degradation kinetics (Table IV). The linear fitting results revealed that the degradation kinetics of rapamycin fitted the first-order model.

**Figure 3:**
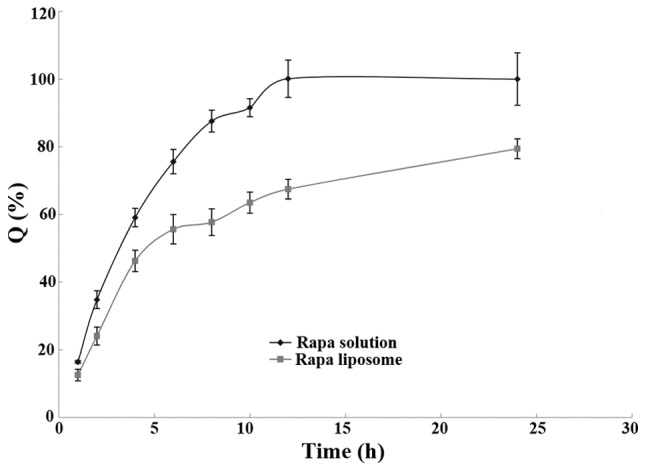
Fitting release curve of rapamycin (rapa) solution and liposomes *in vitro*. Q, accumulative release rate.

The amount of released drug at each time point was entered into the fitted degradation kinetics equation to calculate the amount of degraded drug; the accumulation of the released and degraded drug was set as the ordinate and the time t was set as the abscissa to fit the release profile of the rapamycin-containing liposomes. The resulting curve is shown in Fig. 3.

As can be observed in Fig. 3, the liposomes had a sustained-release effect for rapamycin; the 24 h total release dose was 80%. The release process of rapamycin from the liposomes can be divided into two phases: In the first 4 h, the release rate was fast and ~50% of the total dose was released; this phase was the rapid release phase. This may be due to the release of the encapsulated free drug and the drug that was adsorbed on the surface of the liposomes by a weak binding force. Four hours later, the drug release became relatively slow, known as the slow release phase. The rapamycin solution and rapamycin-containing liposomes were fitted according to the release models including the Higuchi (11) Ritger-Peppas (12) and Weibull (13) models; the results are shown in Tables V and VI. The results indicated that the *in vitro* release profile of rapamycin from the liposomes and solution best fitted the first-order release model.

## Discussion

With the use of new technology and equipment, and the development and application of excellent carrier materials and accessories, targeted drug delivery (TDD) technology has been developing rapidly in recent years, and has gradually extended to the treatment fields of multiple diseases (14–17).

Although nanoparticles, nanocapsules, microspheres, microcapsule, micelle multimers and monoclonal antibody coupling may be considered as the ideal medicament carriers, liposomes remain inexpensive, readily available and more studied drug carriers (18–21).

Liposomes are extensively researched TDD carriers, with a sustained-release, long-lasting effect, low systemic toxicity and good biocompatibility, and are suitable for administration by a variety of routes. However, traditional liposomes only have a passive targeting role; if antibody or ligand molecules, which are targeted towards proliferated intimal tissues, are connected to the liposomal membrane and thus form targeting liposomes, active targeting can be achieved, significantly enhancing the concentration of liposomes in vascular tissues, particularly in atherosclerotic plaques (22–26). The liposomal bilayer is amenable to surface modification to achieve TDD towards the cardiovascular system.

Rapamycin is a lipophilic substance, which is easily compatible with the hydrophobic chains that are used in the preparation of liposomes, and form a part of the bilayer. The optimum mass ratio of phospholipids, cholesterol and rapamycin is that at which liposomes with the highest EE are formed. In this study, the ethanol injection method was used to prepare rapamycin-containing liposomes, a microcolumn centrifugation HPLC method was used to determine the EE, and the EE was used as the evaluation indicator to inspect the impacts of phospholipid concentration, phospholipid-cholesterol mass ratio, drug-lipid ratio and aqueous phase pH on the liposomes. Based on these evaluations, an orthogonal design experiment was performed to optimize the formulation. The orthogonal test results revealed that the best formulation had a phospholipid concentration of 4%, phospholipid-cholesterol mass ratio of 8:1, drug-phospholipid mass ratio of 1:20 and aqueous phase pH of 7.4. The encapsulation rate of the resultant rapamycin-containing liposomes was high, reaching 82.11±2.13%, and the reproducibility was good. The higher EE also indicated that the lipid bilayer was able to significantly solubilize the hydrophobic drug rapamycin, enabling it to be administered through the inner vessel, which would be an effective means to resolve the low oral bioavailability of rapamycin.

The EE is an important indicator when evaluating a liposomal delivery system, and there are numerous methods for determining it. In this study, Sephadex column chromatography was initially used, with online elution using a buffer of pH 7.4; however, free rapamycin did not dissolve in the buffer and could not be eluted quickly. Subsequently, a dialysis method was used to isolate the liposomes and free the drug. As rapamycin is strongly liposoluble, the unencapsulated drug was present in the external aqueous phase in the form of small crystals, which would not pass through the dialysis bag; therefore, the measured quantity of free drug was likely to be inaccurate. Finally, with reference to the literature, a microcolumn centrifugation HPLC method was established to determine the EE; the method was simple and reproducible.

For drugs with poor solubility, when performing a release characteristics study, a surfactant or an organic solvent is typically used to improve the drug solubility in the release medium to meet sink conditions. In the present study, ethanol was selected as a cosolvent to increase the solubility of rapamycin. When the rapamycin-containing liposomes reached sink conditions, drug release was not complete in 24 h, indicating that the liposomes had sustained release effects.

Currently, the main method used in *in vitro* release studies of liposomal preparations is the dialysis method (27). In this method, liposomes contact only a small amount of release medium in the dialysis bag; when the drug diffuses from the liposomes and is released into the release medium in the bag, it then diffuses through the dialysis bag to a massive release medium. As the drug concentration gradient inside and outside the dialysis bag is small, passive diffusion is slow, so that the release rate is low. In the present study, the reverse dialysis method was used to determine the *in vitro* release of rapamycin from the liposomes (9); since the liposomes directly contacted the release medium and were largely diluted, it better simulated the state that the drug achieves following intravenous injection. The experimental results indicated that the dialysis bag had almost no adsorption of rapamycin, and the rapamycin recovery rate was 99.21±1.12% (n=3).

In order to investigate the release properties of the rapamycin-containing liposomes, a release equation was used for fitting, and the optimal equation was determined by means of the correlation coefficient r. The results revealed that the release of rapamycin from the solution and liposomes best fitted the first-order release model, in which the *in vitro* release may be described by a concentration-dependent permeation release model. Since the *in vitro* release conditions were not the same as the *in vivo* blood environment, the *in vitro* release experiment did not truly reflect the situation of *in vivo* release. Further studies of the correlation of *in vivo* absorption and *in vitro* release of rapamycin from the liposomal delivery system are required.

## Figures and Tables

**Table I:** Factors and levels.

	Factors			
	
Levels	A	B	C	D
Level 1	2	10:1	1:15	7.4
Level 2	3	8:1	1:20	7.0
Level 3	4	6:1	1:30	6.5

A, phospholipid concentration; B, phospholipid-cholesterol mass ratio; C, drug-lipid ratio; D, aqueous phase pH.

**Table II:** Results of orthogonal testing.

No.	A	B	C	D	EE %
1	1	1	1	1	71.75
2	1	2	2	2	74.64
3	1	3	3	3	72.80
4	2	1	2	3	75.61
5	2	2	3	1	81.16
6	2	3	1	2	70.49
7	3	1	3	2	85.64
8	3	2	1	3	77.39
9	3	3	2	1	84.28
K_1_	219.19	233.00	219.63	237.19	
K_2_	227.26	233.19	234.53	230.77	
K_3_	247.31	227.57	239.60	225.80	
K̄_1_	73.06	77.67	73.21	79.06	
K̄_2_	75.75	77.73	78.18	76.92	
K̄_3_	82.44	75.86	79.87	75.27	
R	9.38	1.87	6.66	3.79	

A, phospholipid concentration; B, phospholipid-cholesterol mass ratio; C, drug-lipid ratio; D, aqueous phase pH; EE, encapsulation efficiency; K_n_, sum of EE value at level n; R, range (R = K_max_ - K_min_); K̄_n_, average value of K_n_.

**Table III:** Variance analysis.

Source	S	f	MS	F-statistic	P-value
Factor A	139.98	2	69.99	635.27	<0.01
Factor B	6.78	2	3.39	30.82	<0.05
Factor C	71.92	2	35.96	326.91	<0.01
Factor D	21.67	2	10.84	98.55	<0.01
Error	0.22	2	0.11		

S, sum of deviation square; f, degree of freedom; MS, mean square; A, phospholipid concentration; B, phospholipid-cholesterol mass ratio; C, drug-lipid ratio; D, aqueous phase pH. F_0.05_ (2,2)=19; F_0.01_ (2,2)=99.

**Table IV:** Degradation curve-fitting equations of rapamycin solution in release medium.

Model	Fitting equation	Correlation coefficient r
Zero-order model	Cr = −0.0122 t + 0.9397	0.9786
First order model	lnCr = −0.0162 t + 0.0095	0.9931
Second order model	1/Cr = 0.0202 t + 0.9711	0.9809

Cr, residual percentage; t, time.

**Table V:** Release curve-fitting equations of rapamycin solution.

Model	Fitting equation	Correlation coefficient r
Zero-order release model	Q = 0.073 t + 0.2158	0.9181
First-order release model	ln(1-Q) = −0.2609 t + 0.1046	0.9959
Higuchi model	Q = 0.3402 t^1/2^ − 0.1289	0.9802
Ritger-Peppas model	lnQ = 0.7144 lnt − 1.6521	0.9076
Weibull model	ln(1/1-Q) = 0.9816 lnt − 0.1067	0.9003

Q, accumulative release rate; t, time.

**Table VI:** Release curve-fitting equations of rapamycin-containing liposomes.

Model	Fitting equation	Correlation coefficient r
Zero-order release model	Q = 0.0152 t + 0.4544	0.9101
First-order release model	ln(1-Q) = −0.046 t − 0.5090	0.9770
Higuchi model	Q = 0.1166 t^1/2^ + 0.2697	0.9673
Ritger-Peppas model	lnQ = 0.2965 lnt − 0.1067	0.9600
Weibull model	ln(1/1-Q) = 0.5311 lnt − 0.1704	0.9710

Q, accumulative release rate; t, time.

## References

[1] de Jager SC, Kuiper J (2011). Vaccination strategies in atherosclerosis. Thromb Haemost.

[2] Sabers CJ, Martin MM, Brunn GJ, Williams JM, Dumont FJ, Wiederrecht G, Abraham RT (1994). Isolation of a protein target of the FKBP12-rapamycin complex in mammalian cells. J Biol Chem.

[3] Cheng-Lai A, Frishman WH (2004). Sirolimus-eluting coronary stents: novel devices for the management of coronary artery disease. Am J Ther.

[4] Klugherz BD, Llanos G, Lieuallen W (2000). Stent-based delivery of sirolimus for the prevention of resrenosis. J Am Coll Cardiol.

[5] Gallo R, Padurean A (1999). Inhibition of intimal thickening after balloon angioplasty in porcine coronary arteries by targeting regulators of the cell cycle. Circulation.

[6] Zhang JA, Anyarambhatla G, Ma L, Ugwu S, Xuan T, Sardone T, Ahmad I (2005). Development and characterization of a novel Cremophor EL free liposome-based paclitaxel (LEP-ETU) formulation. Eur J Pharm Biopharm.

[7] Li C, Deng Y (2004). A novel method for the preparation of liposomes: freeze drying of monophase solutions. J Pharm Sci.

[8] Lu YP, Lasne C, Chouroulinkov I (1986). Use of an orthogonal design method to study two-stage chemical carcinogenesis in BALB/3T3 cells. Carcinogenesis.

[9] Levy MY, Benita S (1990). Drug release from submicronized O/W emulsion: a new in vitro kinetic evaluation model. Int J Pharm.

[10] Rouf MA, Bilensoy E, Vural I, Hıncal AA (2007). Determination of stability of sirolimus following exposure to different conditions. Eur J Pharm Sci.

[11] Higuchi WI, Tzeng CS, Chang SJ, Chiang HJ, Liu CL (2008). Estimation of cholesterol solubilization by a mixed micelle binding model in aqueous tauroursodeoxycholate:lecithin:cholesterol solutions. J Pharm Sci.

[12] Ravi PR, Ganga S, Saha RN (2008). Design and in vitro evaluation of zidovudine oral controlled release tablets prepared using hydroxypropyl methylcellulose. Chem Pharm Bull (Tokyo).

[13] Lionberger RA, Raw AS, Kim SH, Zhang X, Yu LX (2012). Use of partial AUC to demonstrate bioequivalence of Zolpidem Tartrate Extended Release formulations. Pharm Res.

[14] Masaka T, Matsuda T, Li Y (2013). Synthesis of VIP-lipopeptide using a new linker to modify liposomes: Towards the development of a drug delivery system for active targeting. Chem Pharm Bull (Tokyo).

[15] Wang X, Lin Y, Zeng Y, Sun X, Gong T, Zhang Z (2013). Effects of mycophenolic acid-glucosamine conjugates on the base of kidney targeted drug delivery. Int J Pharm.

[16] Pehlivan SB (2013). Nanotechnology-based drug delivery systems for targeting, imaging and diagnosis of neurodegenerative diseases. Pharm Res.

[17] Arias JL (2013). Liposomes in drug delivery: a patent review (2007 - present). Expert Opin Ther Pat.

[18] Murakami M, Cabral H, Matsumoto Y (2011). Improving drug potency and efficacy by nanocarrier-mediated subcellular targeting. Sci Transl Med.

[19] Musacchio T, Torchilin VP (2011). Recent developments in lipid-based pharmaceutical nanocarriers. Front Biosci (Landmark Ed).

[20] Roger M, Clavreul A, Venier-Julienne MC, Passirani C, Montero-Menei C, Menei P (2011). The potential of combinations of drug-loaded nanoparticle systems and adult stem cells for glioma therapy. Biomaterials.

[21] Suo X, Deng Y, Hao A (2005). Determination of lauroyl-indapamide in rat whole blood by high-performance liquid chromatography. J Chromatogr B Analyt Technol Biomed Life Sci.

[22] Torchilin VP, Narula J, Halpern E, Khaw BA (1996). Poly(ethylene glycol)-coated anti-cardiac myosin immunoliposomes: factors influencing targeted accumulation in the infarcted myocardlium. Biochem Biophys Acta.

[23] Chen Y, Deng YJ, Hao YL (2005). Surface modification of liposomes for cardiomyocytes targeting in vitro. Pharmazie.

[24] Chen Y, Deng YJ, Hao YL, Hao AJ, Zhong HJ, Wang XM (2005). Uptake of liposomes by cultured cardiomyocytes. Pharmazie.

[25] Nag OK, Awasthi V (2013). Surface engineering of liposomes for stealth behavior. Pharmaceutics.

[26] Paszko E, Senge MO (2012). Immunoliposomes. Curr Med Chem.

[27] Shen J, Burgess DJ (2013). In vitro dissolution testing strategies for nanoparticulate drug delivery systems: recent developments and challenges. Drug Deliv Transl Res.

